# Ethyl 6-(4-fluoro­phen­yl)-4-hy­droxy-2-oxo-4-trifluoro­meth­yl-1,3-diazinane-5-carboxyl­ate monohydrate

**DOI:** 10.1107/S1600536811021866

**Published:** 2011-06-18

**Authors:** Gong-Chun Li, Chang-Zeng Wu, Li-Li Guo, Feng-Ling Yang

**Affiliations:** a College of Chemistry and Chemical Engineering, Xuchang University, Xuchang, Henan Province 461000, People’s Republic of China; b Department of Chemistry, Zhengzhou University, Zhengzhou Henan Province, 450052, People’s Republic of China

## Abstract

The asymmetric unit of the title compound, C_14_H_14_F_4_N_2_O_4_·H_2_O, contains two crystallographically independent organic mol­ecules and two water mol­ecules. The two 1,3-diazinane rings adopt a half-chair conformation and the dihedral angles between their mean planes and those of the benzene rings are 75.65 (4)° and 49.41 (3)° in the two mol­ecules. The crystal structure is stabilized by inter­molecular O—H⋯O and N—H⋯O hydrogen bonds.

## Related literature

For the bioactivity of dihydro­pyrimidines, see: Brier *et al.* (2004 ▶); Cochran *et al.* (2005 ▶); Moran *et al.* (2007 ▶); Zorkun *et al.* (2006 ▶). For the bioactivity of organofluorine compounds, see: Hermann *et al.* (2003 ▶); Ulrich (2004 ▶). For a related structure, see: Song *et al.* (2010 ▶).
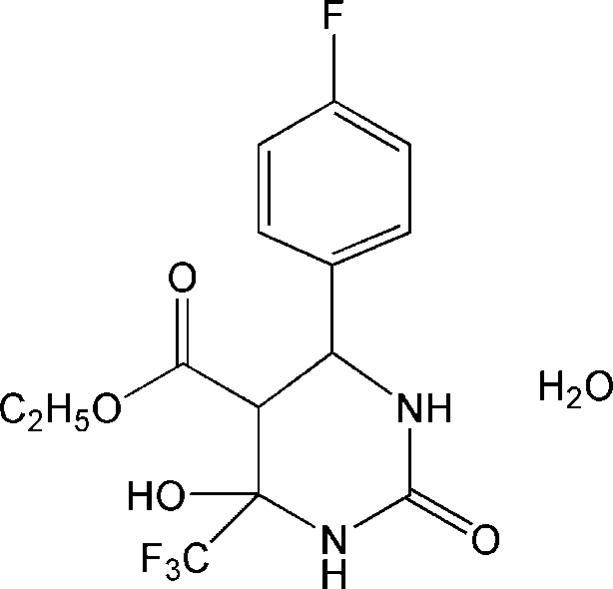


## Experimental

### 

#### Crystal data

C_14_H_14_F_4_N_2_O_4_·H_2_O
*M* *_r_* = 368.29
Triclinic, 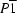

*a* = 10.0196 (9) Å
*b* = 12.1718 (12) Å
*c* = 14.3037 (14) Å
α = 98.463 (7)°
β = 103.642 (8)°
γ = 104.400 (9)°
*V* = 1602.2 (3) Å^3^
*Z* = 4
Mo *K*α radiation
μ = 0.14 mm^−1^
*T* = 113 K
0.20 × 0.18 × 0.14 mm

#### Data collection

Rigaku Saturn CCD area detector diffractometer
Absorption correction: multi-scan (*CrystalClear*; Rigaku, 2009 ▶) *T*_min_ = 0.972, *T*_max_ = 0.980
20692 measured reflections
7612 independent reflections
4575 reflections with *I* > 2σ(*I*)
*R*_int_ = 0.041

#### Refinement

*R*[*F* ^2^ > 2σ(*F* ^2^)] = 0.031
*wR*(*F* ^2^) = 0.072
*S* = 0.87
7612 reflections
493 parameters
H atoms treated by a mixture of independent and constrained refinement
Δρ_max_ = 0.35 e Å^−3^
Δρ_min_ = −0.19 e Å^−3^

### 

Data collection: *CrystalClear* (Rigaku, 2009 ▶); cell refinement: *CrystalClear*; data reduction: *CrystalClear*; program(s) used to solve structure: *SHELXS97* (Sheldrick, 2008 ▶); program(s) used to refine structure: *SHELXL97* (Sheldrick, 2008 ▶); molecular graphics: *SHELXTL* (Sheldrick, 2008 ▶); software used to prepare material for publication: *SHELXTL*.

## Supplementary Material

Crystal structure: contains datablock(s) global, I. DOI: 10.1107/S1600536811021866/jh2293sup1.cif

Structure factors: contains datablock(s) I. DOI: 10.1107/S1600536811021866/jh2293Isup2.hkl

Supplementary material file. DOI: 10.1107/S1600536811021866/jh2293Isup3.cml

Additional supplementary materials:  crystallographic information; 3D view; checkCIF report

## Figures and Tables

**Table 1:** Hydrogen-bond geometry (Å, °)

*D*—H⋯*A*	*D*—H	H⋯*A*	*D*⋯*A*	*D*—H⋯*A*
O2—H2⋯O9^i^	0.889 (15)	1.860 (16)	2.7482 (14)	176.2 (15)
O9—H9*A*⋯O3^ii^	0.91 (2)	1.90 (2)	2.8026 (15)	169.8 (18)
O6—H6⋯O10^iii^	0.893 (16)	1.788 (16)	2.6770 (14)	173.5 (15)
N3—H3*A*⋯O10^iii^	0.823 (14)	2.583 (15)	3.0737 (16)	119.6 (12)
N4—H4*A*⋯O5^iii^	0.904 (14)	1.900 (15)	2.8010 (14)	174.8 (13)
N1—H1*A*⋯O1^iv^	0.828 (14)	2.109 (14)	2.9235 (15)	167.9 (13)
N2—H2*A*⋯O5^v^	0.906 (14)	1.958 (15)	2.8395 (14)	163.8 (13)
O9—H9*B*⋯O1^vi^	0.80 (2)	2.00 (2)	2.7443 (15)	155 (2)
O10—H10*A*⋯O9^vii^	0.861 (17)	1.925 (17)	2.7833 (15)	175.4 (16)
O10—H10*B*⋯O7^viii^	0.82 (2)	2.07 (2)	2.8685 (14)	166 (2)

Symmetry codes: (i) 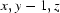; (ii) 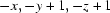; (iii) 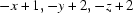; (iv) 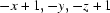; (v) 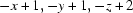; (vi) 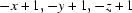; (vii) 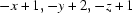; (viii) 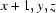.

**Fig. 1.**
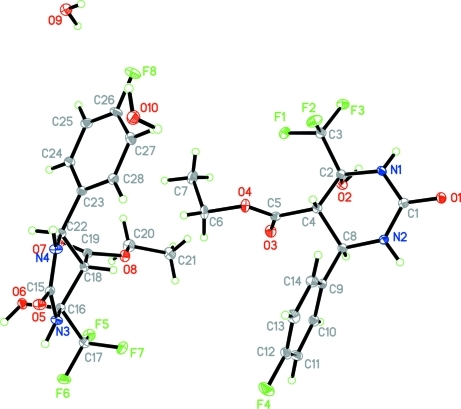
Molecular configuration and atom numbering scheme for (I), with displacement ellipsoids drawn at the 30% probability level.

## supplementary crystallographic information

### Comment

Dihydropyrimidine (DHPM) derivatives can be used as potential calcium channel blockers (Zorkun *et al.*, 2006), inhibitors of mitotic kinesin Eg5 for treating cancer (Cochran *et al.*, 2005; Brier *et al.*, 2004) and as TRPA1 modulators for treating pain (Moran *et al.*, 2007). In addition, compounds that contain fluorine have special bioactivity, *e.g.* flumioxazin is a widely used herbicide (Hermann *et al.*, 2003; Ulrich, 2004). This led us to focus our attention on the synthesis and bioactivity of these important fused perfluoroalkylated heterocyclic compounds. During the synthesis of DHPM derivatives, the title compound, an intermediate C_14_H_14_F_4_N_2_O_4_.H_2_O(I) was isolated and the structure confirmed by X-ray diffraction.

The asymmetric unit of the title compound contains two crystallographically independent organic molecules and two water molecules. The two 1,3-diazinane rings adopt half-chair conformation, the mean planes formed by the ring atoms excluding the C atom bonded to the ethoxy carbonyl group have r.m.s. deviations of 0.0202Å and 0.0397 Å, the dihedral angles between the mean planes and benzenes ring are 75.65 (4)° and 49.41 (3)° respectively. The crystal structure is stabilized by intermolecular hydrogen bonds (O—H···O and N—H···O). For a crystal structure related to the title compound, see: Song *et al.*, 2010.

### Experimental

The title compound was synthesized refluxing for 3 h a stirred solution of 4-fluorobenzaldehyde (2.50 g, 20 mmol), ethyl ethyl 4,4,4-trifluoroacetoacetate (4.42 g, 24 mmol) and urea (1.80 g, 30 mmol) in 20 ml of anhydrous ethanol. The reaction was catalyzed by sulfamic acid (0.6 g). The solvent was evaporated *in vacuo* and the residue was washed with water. The title compound was recrystallized from 50% aqueous ethanol and single crystals of the title compound were obtained by slow evaporation of mother liquor.

### Refinement

Hydrogen atoms involved in hydrogen-bonding inetractions were located by difference methods and their positional and isotropic displacement parameters were refined. Other H atoms were placed in calculated positions, with C—H(aromatic) = 0.95 Å and C—H(aliphatic) = 0.98 Å, 0.99 Å or 1.00 Å, and treated as riding, with *U*_iso_(H) = 1.2*U*eq(C).

### Crystal data

**Table:**

C_14_H_14_F_4_N_2_O_4_·H_2_O	*Z* = 4
*M**_r_* = 368.29	*F*(000) = 760
Triclinic, *P*1	*D*_x_ = 1.527 Mg m^−^^3^
*a* = 10.0196 (9) Å	Mo *K*α radiation, λ = 0.71073 Å
*b* = 12.1718 (12) Å	Cell parameters from 5664 reflections
*c* = 14.3037 (14) Å	θ = 1.5–27.9°
α = 98.463 (7)°	µ = 0.14 mm^−^^1^
β = 103.642 (8)°	*T* = 113 K
γ = 104.400 (9)°	Prism, colorless
*V* = 1602.2 (3) Å^3^	0.20 × 0.18 × 0.14 mm

### Data collection

**Table:**

Rigaku Saturn CCD area detector diffractometer	7612 independent reflections
Radiation source: rotating anode	4575 reflections with *I* > 2σ(*I*)
multilayer	*R*_int_ = 0.041
Detector resolution: 14.63 pixels mm^-1^	θ_max_ = 27.9°, θ_min_ = 1.5°
ω and φ scans	*h* = −13→13
Absorption correction: multi-scan (*CrystalClear*; Rigaku, 2009)	*k* = −16→14
*T*_min_ = 0.972, *T*_max_ = 0.980	*l* = −18→18
20692 measured reflections	

### Refinement

**Table:**

Refinement on *F*^2^	Primary atom site location: structure-invariant direct methods
Least-squares matrix: full	Secondary atom site location: difference Fourier map
*R*[*F*^2^ > 2σ(*F*^2^)] = 0.031	Hydrogen site location: inferred from neighbouring sites
*wR*(*F*^2^) = 0.072	H atoms treated by a mixture of independent and constrained refinement
*S* = 0.87	*w* = 1/[σ^2^(*F*_o_^2^) + (0.0267*P*)^2^] where *P* = (*F*_o_^2^ + 2*F*_c_^2^)/3
7612 reflections	(Δ/σ)_max_ = 0.001
493 parameters	Δρ_max_ = 0.35 e Å^−^^3^
0 restraints	Δρ_min_ = −0.19 e Å^−^^3^

### Special details

**Table:**

Geometry. All e.s.d.'s (except the e.s.d. in the dihedral angle between two l.s. planes) are estimated using the full covariance matrix. The cell e.s.d.'s are taken into account individually in the estimation of e.s.d.'s in distances, angles and torsion angles; correlations between e.s.d.'s in cell parameters are only used when they are defined by crystal symmetry. An approximate (isotropic) treatment of cell e.s.d.'s is used for estimating e.s.d.'s involving l.s. planes.
Refinement. Refinement of *F*^2^ against ALL reflections. The weighted *R*-factor *wR* and goodness of fit *S* are based on *F*^2^, conventional *R*-factors *R* are based on *F*, with *F* set to zero for negative *F*^2^. The threshold expression of *F*^2^ > σ(*F*^2^) is used only for calculating *R*-factors(gt) *etc*. and is not relevant to the choice of reflections for refinement. *R*-factors based on *F*^2^ are statistically about twice as large as those based on *F*, and *R*- factors based on ALL data will be even larger.

### Fractional atomic coordinates and isotropic or equivalent isotropic displacement parameters (Å^2^)

**Table:**

	*x*	*y*	*z*	*U*_iso_*/*U*_eq_	
F1	0.21735 (8)	0.30976 (7)	0.47711 (6)	0.0278 (2)	
F2	0.44636 (9)	0.33996 (7)	0.51405 (6)	0.0313 (2)	
F3	0.30625 (9)	0.19817 (7)	0.39477 (6)	0.0288 (2)	
F4	0.49822 (9)	0.38466 (7)	1.13399 (6)	0.0355 (2)	
F5	−0.11880 (8)	0.62729 (6)	0.98946 (6)	0.02501 (19)	
F6	0.01160 (8)	0.72196 (7)	1.13589 (6)	0.02698 (19)	
F7	0.09316 (8)	0.61081 (6)	1.04636 (6)	0.02592 (19)	
F8	0.28213 (9)	0.78195 (7)	0.49828 (6)	0.0356 (2)	
O1	0.57514 (10)	0.02599 (8)	0.62488 (6)	0.0205 (2)	
O2	0.16764 (10)	0.09767 (8)	0.52602 (7)	0.0212 (2)	
H2	0.1553 (16)	0.0484 (13)	0.4701 (12)	0.041 (5)*	
O3	0.10031 (10)	0.25206 (8)	0.67210 (7)	0.0236 (2)	
O4	0.27857 (9)	0.41634 (7)	0.69581 (7)	0.0218 (2)	
O5	0.44555 (9)	0.97089 (7)	1.10542 (6)	0.0191 (2)	
O6	−0.00739 (10)	0.86354 (8)	0.99826 (7)	0.0189 (2)	
H6	0.0185 (16)	0.9111 (13)	1.0575 (12)	0.039 (5)*	
O7	−0.13754 (10)	0.74745 (8)	0.79365 (7)	0.0226 (2)	
O8	−0.07876 (9)	0.58103 (7)	0.79232 (6)	0.0209 (2)	
N1	0.41809 (12)	0.11768 (10)	0.55571 (8)	0.0196 (3)	
N2	0.45729 (12)	0.10551 (10)	0.71961 (8)	0.0191 (3)	
N3	0.22350 (12)	0.84435 (9)	1.06802 (8)	0.0173 (2)	
N4	0.31320 (12)	0.91025 (10)	0.94499 (8)	0.0184 (3)	
C1	0.48618 (14)	0.07974 (11)	0.63376 (9)	0.0175 (3)	
C2	0.30765 (14)	0.17404 (11)	0.55694 (9)	0.0172 (3)	
C3	0.32023 (15)	0.25634 (12)	0.48542 (10)	0.0211 (3)	
C4	0.33613 (14)	0.24284 (11)	0.66264 (9)	0.0163 (3)	
H4	0.4306	0.3044	0.6806	0.020*	
C5	0.22216 (14)	0.30121 (11)	0.67569 (9)	0.0177 (3)	
C6	0.18167 (15)	0.48463 (12)	0.71134 (10)	0.0256 (3)	
H6A	0.2381	0.5624	0.7530	0.031*	
H6B	0.1156	0.4457	0.7462	0.031*	
C7	0.09575 (15)	0.49743 (12)	0.61357 (10)	0.0292 (4)	
H7A	0.1613	0.5278	0.5760	0.044*	
H7B	0.0402	0.5513	0.6251	0.044*	
H7C	0.0301	0.4214	0.5762	0.044*	
C8	0.34703 (14)	0.16004 (11)	0.73406 (9)	0.0167 (3)	
H8	0.2527	0.0986	0.7177	0.020*	
C9	0.38729 (14)	0.22261 (11)	0.84106 (9)	0.0169 (3)	
C10	0.30035 (14)	0.18702 (11)	0.90023 (10)	0.0198 (3)	
H10	0.2135	0.1254	0.8723	0.024*	
C11	0.33834 (15)	0.23995 (12)	0.99955 (10)	0.0232 (3)	
H11	0.2798	0.2143	1.0402	0.028*	
C12	0.46237 (15)	0.32993 (12)	1.03707 (10)	0.0235 (3)	
C13	0.55181 (15)	0.36883 (12)	0.98175 (10)	0.0259 (3)	
H13	0.6374	0.4316	1.0101	0.031*	
C14	0.51319 (15)	0.31362 (12)	0.88317 (10)	0.0231 (3)	
H14	0.5740	0.3385	0.8437	0.028*	
C15	0.33267 (14)	0.91286 (11)	1.04127 (9)	0.0164 (3)	
C16	0.08059 (13)	0.79019 (11)	1.00238 (9)	0.0157 (3)	
C17	0.01571 (14)	0.68607 (11)	1.04325 (10)	0.0196 (3)	
C18	0.09283 (13)	0.75018 (11)	0.89773 (9)	0.0157 (3)	
H18	0.1481	0.6921	0.8994	0.019*	
C19	−0.05367 (14)	0.69559 (11)	0.82313 (9)	0.0179 (3)	
C20	−0.21972 (15)	0.51967 (11)	0.72276 (10)	0.0263 (3)	
H20A	−0.2292	0.5474	0.6605	0.032*	
H20B	−0.2968	0.5335	0.7510	0.032*	
C21	−0.23104 (15)	0.39215 (11)	0.70373 (10)	0.0267 (3)	
H21A	−0.1517	0.3801	0.6785	0.040*	
H21B	−0.3228	0.3484	0.6550	0.040*	
H21C	−0.2259	0.3650	0.7653	0.040*	
C22	0.17640 (13)	0.85802 (11)	0.86849 (9)	0.0160 (3)	
H22	0.1201	0.9153	0.8673	0.019*	
C23	0.20333 (14)	0.83296 (10)	0.76879 (9)	0.0162 (3)	
C24	0.10992 (14)	0.84699 (11)	0.68548 (9)	0.0197 (3)	
H24	0.0276	0.8695	0.6915	0.024*	
C25	0.13467 (15)	0.82869 (11)	0.59370 (10)	0.0218 (3)	
H25	0.0699	0.8373	0.5369	0.026*	
C26	0.25512 (15)	0.79791 (11)	0.58763 (10)	0.0233 (3)	
C27	0.35117 (15)	0.78274 (11)	0.66745 (10)	0.0225 (3)	
H27	0.4336	0.7610	0.6604	0.027*	
C28	0.32418 (14)	0.80009 (11)	0.75858 (10)	0.0198 (3)	
H28	0.3886	0.7895	0.8146	0.024*	
O9	0.13823 (13)	0.94116 (9)	0.35763 (7)	0.0243 (2)	
O10	0.90924 (12)	0.99386 (10)	0.82314 (8)	0.0282 (3)	
H1A	0.4218 (15)	0.0862 (12)	0.5014 (10)	0.027 (4)*	
H2A	0.4935 (15)	0.0714 (12)	0.7678 (11)	0.035 (4)*	
H3A	0.2281 (16)	0.8644 (13)	1.1265 (11)	0.033 (5)*	
H4A	0.3906 (15)	0.9527 (12)	0.9307 (10)	0.032 (4)*	
H9A	0.065 (2)	0.8778 (17)	0.3556 (14)	0.081 (7)*	
H9B	0.215 (2)	0.9295 (17)	0.3632 (14)	0.074 (8)*	
H10A	0.8938 (18)	1.0100 (14)	0.7657 (13)	0.049 (5)*	
H10B	0.883 (2)	0.9233 (18)	0.8184 (14)	0.080 (8)*	

### Atomic displacement parameters (Å^2^)

**Table:**

	*U*^11^	*U*^22^	*U*^33^	*U*^12^	*U*^13^	*U*^23^
F1	0.0324 (5)	0.0316 (5)	0.0278 (5)	0.0196 (4)	0.0087 (4)	0.0144 (4)
F2	0.0268 (5)	0.0318 (5)	0.0332 (5)	0.0016 (4)	0.0064 (4)	0.0162 (4)
F3	0.0391 (5)	0.0352 (5)	0.0176 (4)	0.0168 (4)	0.0097 (4)	0.0099 (4)
F4	0.0378 (5)	0.0467 (5)	0.0169 (4)	0.0151 (4)	0.0028 (4)	−0.0048 (4)
F5	0.0194 (4)	0.0231 (4)	0.0288 (5)	0.0000 (4)	0.0064 (4)	0.0057 (4)
F6	0.0336 (5)	0.0287 (4)	0.0203 (4)	0.0055 (4)	0.0132 (4)	0.0077 (4)
F7	0.0281 (5)	0.0213 (4)	0.0317 (5)	0.0099 (4)	0.0087 (4)	0.0108 (4)
F8	0.0433 (5)	0.0454 (5)	0.0210 (5)	0.0118 (5)	0.0179 (4)	0.0039 (4)
O1	0.0218 (5)	0.0253 (5)	0.0187 (5)	0.0129 (4)	0.0065 (4)	0.0059 (4)
O2	0.0193 (5)	0.0221 (5)	0.0186 (5)	0.0038 (4)	0.0036 (4)	0.0002 (4)
O3	0.0181 (5)	0.0226 (5)	0.0303 (6)	0.0047 (4)	0.0091 (4)	0.0047 (4)
O4	0.0203 (5)	0.0157 (5)	0.0286 (6)	0.0071 (4)	0.0053 (4)	0.0021 (4)
O5	0.0160 (5)	0.0221 (5)	0.0142 (5)	0.0011 (4)	0.0005 (4)	0.0029 (4)
O6	0.0198 (5)	0.0197 (5)	0.0186 (5)	0.0091 (4)	0.0056 (4)	0.0024 (4)
O7	0.0203 (5)	0.0217 (5)	0.0233 (5)	0.0066 (4)	0.0013 (4)	0.0051 (4)
O8	0.0182 (5)	0.0172 (5)	0.0213 (5)	0.0027 (4)	−0.0001 (4)	−0.0008 (4)
N1	0.0239 (7)	0.0268 (6)	0.0136 (6)	0.0150 (5)	0.0070 (5)	0.0057 (5)
N2	0.0240 (7)	0.0236 (6)	0.0144 (6)	0.0136 (5)	0.0059 (5)	0.0067 (5)
N3	0.0170 (6)	0.0212 (6)	0.0120 (6)	0.0030 (5)	0.0039 (5)	0.0029 (5)
N4	0.0141 (6)	0.0227 (6)	0.0144 (6)	0.0000 (5)	0.0019 (5)	0.0044 (5)
C1	0.0184 (7)	0.0163 (7)	0.0162 (7)	0.0035 (6)	0.0039 (6)	0.0035 (5)
C2	0.0157 (7)	0.0191 (7)	0.0173 (7)	0.0062 (6)	0.0040 (6)	0.0043 (6)
C3	0.0199 (8)	0.0248 (7)	0.0201 (7)	0.0090 (6)	0.0048 (6)	0.0070 (6)
C4	0.0147 (7)	0.0182 (7)	0.0152 (7)	0.0048 (6)	0.0025 (6)	0.0035 (5)
C5	0.0205 (8)	0.0190 (7)	0.0133 (7)	0.0066 (6)	0.0028 (6)	0.0039 (5)
C6	0.0258 (8)	0.0189 (7)	0.0319 (8)	0.0124 (6)	0.0054 (7)	−0.0003 (6)
C7	0.0308 (9)	0.0266 (8)	0.0327 (9)	0.0143 (7)	0.0064 (7)	0.0085 (7)
C8	0.0169 (7)	0.0180 (7)	0.0158 (7)	0.0060 (6)	0.0047 (6)	0.0036 (5)
C9	0.0182 (7)	0.0180 (7)	0.0172 (7)	0.0096 (6)	0.0047 (6)	0.0051 (5)
C10	0.0179 (7)	0.0221 (7)	0.0196 (7)	0.0074 (6)	0.0047 (6)	0.0037 (6)
C11	0.0225 (8)	0.0327 (8)	0.0193 (7)	0.0127 (7)	0.0090 (6)	0.0079 (6)
C12	0.0260 (8)	0.0299 (8)	0.0145 (7)	0.0148 (7)	0.0015 (6)	−0.0002 (6)
C13	0.0215 (8)	0.0250 (8)	0.0244 (8)	0.0030 (6)	0.0016 (6)	−0.0007 (6)
C14	0.0218 (8)	0.0253 (7)	0.0223 (8)	0.0051 (6)	0.0084 (6)	0.0057 (6)
C15	0.0180 (7)	0.0151 (6)	0.0171 (7)	0.0062 (6)	0.0044 (6)	0.0048 (5)
C16	0.0140 (7)	0.0167 (6)	0.0154 (7)	0.0043 (6)	0.0030 (6)	0.0034 (5)
C17	0.0189 (8)	0.0210 (7)	0.0186 (7)	0.0055 (6)	0.0058 (6)	0.0033 (6)
C18	0.0147 (7)	0.0158 (6)	0.0160 (7)	0.0048 (6)	0.0036 (6)	0.0025 (5)
C19	0.0190 (7)	0.0180 (7)	0.0163 (7)	0.0034 (6)	0.0066 (6)	0.0034 (6)
C20	0.0190 (8)	0.0252 (8)	0.0249 (8)	0.0007 (6)	−0.0027 (6)	−0.0001 (6)
C21	0.0211 (8)	0.0247 (8)	0.0265 (8)	0.0003 (6)	0.0034 (7)	−0.0017 (6)
C22	0.0145 (7)	0.0168 (6)	0.0142 (7)	0.0029 (6)	0.0014 (6)	0.0024 (5)
C23	0.0183 (7)	0.0142 (6)	0.0146 (7)	0.0018 (6)	0.0054 (6)	0.0025 (5)
C24	0.0182 (7)	0.0228 (7)	0.0184 (7)	0.0070 (6)	0.0045 (6)	0.0045 (6)
C25	0.0246 (8)	0.0233 (7)	0.0152 (7)	0.0051 (6)	0.0029 (6)	0.0046 (6)
C26	0.0301 (8)	0.0217 (7)	0.0147 (7)	0.0002 (6)	0.0109 (6)	−0.0005 (6)
C27	0.0204 (8)	0.0211 (7)	0.0265 (8)	0.0066 (6)	0.0092 (6)	0.0017 (6)
C28	0.0203 (8)	0.0179 (7)	0.0187 (7)	0.0042 (6)	0.0031 (6)	0.0031 (6)
O9	0.0231 (6)	0.0241 (6)	0.0235 (6)	0.0053 (5)	0.0048 (5)	0.0044 (4)
O10	0.0423 (7)	0.0230 (6)	0.0188 (6)	0.0127 (5)	0.0053 (5)	0.0031 (5)

### Geometric parameters (Å, °)

**Table:**

F1—C3	1.3408 (15)	C7—H7B	0.9800
F2—C3	1.3395 (15)	C7—H7C	0.9800
F3—C3	1.3405 (15)	C8—C9	1.5159 (17)
F4—C12	1.3671 (15)	C8—H8	1.0000
F5—C17	1.3378 (15)	C9—C10	1.3896 (17)
F6—C17	1.3464 (14)	C9—C14	1.3897 (18)
F7—C17	1.3388 (14)	C10—C11	1.3898 (18)
F8—C26	1.3641 (14)	C10—H10	0.9500
O1—C1	1.2480 (15)	C11—C12	1.3666 (19)
O2—C2	1.4019 (15)	C11—H11	0.9500
O2—H2	0.889 (15)	C12—C13	1.374 (2)
O3—C5	1.2039 (15)	C13—C14	1.3871 (18)
O4—C5	1.3328 (15)	C13—H13	0.9500
O4—C6	1.4600 (15)	C14—H14	0.9500
O5—C15	1.2419 (15)	C16—C17	1.5356 (18)
O6—C16	1.4001 (15)	C16—C18	1.5474 (17)
O6—H6	0.893 (16)	C18—C19	1.5173 (18)
O7—C19	1.2072 (15)	C18—C22	1.5424 (17)
O8—C19	1.3395 (15)	C18—H18	1.0000
O8—C20	1.4615 (16)	C20—C21	1.5066 (18)
N1—C1	1.3690 (16)	C20—H20A	0.9900
N1—C2	1.4418 (16)	C20—H20B	0.9900
N1—H1A	0.828 (14)	C21—H21A	0.9800
N2—C1	1.3377 (16)	C21—H21B	0.9800
N2—C8	1.4614 (16)	C21—H21C	0.9800
N2—H2A	0.906 (14)	C22—C23	1.5146 (17)
N3—C15	1.3697 (16)	C22—H22	1.0000
N3—C16	1.4424 (17)	C23—C24	1.3923 (17)
N3—H3A	0.823 (14)	C23—C28	1.3981 (18)
N4—C15	1.3390 (16)	C24—C25	1.3890 (17)
N4—C22	1.4610 (16)	C24—H24	0.9500
N4—H4A	0.904 (14)	C25—C26	1.3671 (19)
C2—C3	1.5380 (18)	C25—H25	0.9500
C2—C4	1.5405 (17)	C26—C27	1.3769 (18)
C4—C5	1.5194 (17)	C27—C28	1.3894 (18)
C4—C8	1.5416 (17)	C27—H27	0.9500
C4—H4	1.0000	C28—H28	0.9500
C6—C7	1.5114 (18)	O9—H9A	0.91 (2)
C6—H6A	0.9900	O9—H9B	0.80 (2)
C6—H6B	0.9900	O10—H10A	0.861 (17)
C7—H7A	0.9800	O10—H10B	0.82 (2)
			
C2—O2—H2	109.7 (10)	F4—C12—C13	118.48 (13)
C5—O4—C6	116.13 (10)	C12—C13—C14	117.91 (13)
C16—O6—H6	107.7 (10)	C12—C13—H13	121.0
C19—O8—C20	115.41 (10)	C14—C13—H13	121.0
C1—N1—C2	124.52 (12)	C13—C14—C9	121.27 (13)
C1—N1—H1A	114.5 (10)	C13—C14—H14	119.4
C2—N1—H1A	117.6 (10)	C9—C14—H14	119.4
C1—N2—C8	124.97 (11)	O5—C15—N4	123.03 (12)
C1—N2—H2A	116.4 (9)	O5—C15—N3	119.63 (12)
C8—N2—H2A	117.0 (9)	N4—C15—N3	117.30 (12)
C15—N3—C16	123.46 (11)	O6—C16—N3	113.49 (10)
C15—N3—H3A	113.1 (11)	O6—C16—C17	109.05 (10)
C16—N3—H3A	115.5 (11)	N3—C16—C17	105.57 (10)
C15—N4—C22	125.04 (11)	O6—C16—C18	109.01 (10)
C15—N4—H4A	113.9 (9)	N3—C16—C18	108.46 (10)
C22—N4—H4A	120.5 (9)	C17—C16—C18	111.26 (11)
O1—C1—N2	121.75 (12)	F5—C17—F7	107.47 (10)
O1—C1—N1	120.47 (12)	F5—C17—F6	107.27 (10)
N2—C1—N1	117.75 (12)	F7—C17—F6	107.20 (10)
O2—C2—N1	114.18 (11)	F5—C17—C16	112.27 (11)
O2—C2—C3	108.30 (10)	F7—C17—C16	111.87 (11)
N1—C2—C3	106.15 (11)	F6—C17—C16	110.49 (11)
O2—C2—C4	108.86 (10)	C19—C18—C22	110.15 (10)
N1—C2—C4	108.53 (10)	C19—C18—C16	111.83 (10)
C3—C2—C4	110.82 (11)	C22—C18—C16	107.50 (10)
F2—C3—F3	107.29 (11)	C19—C18—H18	109.1
F2—C3—F1	106.81 (11)	C22—C18—H18	109.1
F3—C3—F1	107.09 (10)	C16—C18—H18	109.1
F2—C3—C2	112.82 (11)	O7—C19—O8	123.52 (12)
F3—C3—C2	111.21 (11)	O7—C19—C18	125.21 (12)
F1—C3—C2	111.32 (11)	O8—C19—C18	111.27 (11)
C5—C4—C2	114.01 (10)	O8—C20—C21	107.16 (11)
C5—C4—C8	109.05 (11)	O8—C20—H20A	110.3
C2—C4—C8	108.95 (10)	C21—C20—H20A	110.3
C5—C4—H4	108.2	O8—C20—H20B	110.3
C2—C4—H4	108.2	C21—C20—H20B	110.3
C8—C4—H4	108.2	H20A—C20—H20B	108.5
O3—C5—O4	124.46 (12)	C20—C21—H21A	109.5
O3—C5—C4	125.69 (12)	C20—C21—H21B	109.5
O4—C5—C4	109.81 (11)	H21A—C21—H21B	109.5
O4—C6—C7	110.18 (11)	C20—C21—H21C	109.5
O4—C6—H6A	109.6	H21A—C21—H21C	109.5
C7—C6—H6A	109.6	H21B—C21—H21C	109.5
O4—C6—H6B	109.6	N4—C22—C23	109.79 (11)
C7—C6—H6B	109.6	N4—C22—C18	107.87 (10)
H6A—C6—H6B	108.1	C23—C22—C18	114.33 (10)
C6—C7—H7A	109.5	N4—C22—H22	108.2
C6—C7—H7B	109.5	C23—C22—H22	108.2
H7A—C7—H7B	109.5	C18—C22—H22	108.2
C6—C7—H7C	109.5	C24—C23—C28	118.56 (12)
H7A—C7—H7C	109.5	C24—C23—C22	120.04 (11)
H7B—C7—H7C	109.5	C28—C23—C22	121.35 (11)
N2—C8—C9	109.11 (10)	C25—C24—C23	121.33 (12)
N2—C8—C4	107.61 (10)	C25—C24—H24	119.3
C9—C8—C4	112.55 (10)	C23—C24—H24	119.3
N2—C8—H8	109.2	C26—C25—C24	117.96 (12)
C9—C8—H8	109.2	C26—C25—H25	121.0
C4—C8—H8	109.2	C24—C25—H25	121.0
C10—C9—C14	118.47 (13)	F8—C26—C25	118.52 (12)
C10—C9—C8	120.19 (12)	F8—C26—C27	118.24 (12)
C14—C9—C8	121.31 (12)	C25—C26—C27	123.24 (13)
C9—C10—C11	121.14 (13)	C26—C27—C28	118.16 (13)
C9—C10—H10	119.4	C26—C27—H27	120.9
C11—C10—H10	119.4	C28—C27—H27	120.9
C12—C11—C10	118.08 (13)	C27—C28—C23	120.75 (12)
C12—C11—H11	121.0	C27—C28—H28	119.6
C10—C11—H11	121.0	C23—C28—H28	119.6
C11—C12—F4	118.40 (13)	H9A—O9—H9B	114.7 (18)
C11—C12—C13	123.12 (13)	H10A—O10—H10B	110.6 (17)
			
C8—N2—C1—O1	−175.03 (12)	C22—N4—C15—O5	−170.31 (12)
C8—N2—C1—N1	7.05 (19)	C22—N4—C15—N3	12.12 (18)
C2—N1—C1—O1	176.03 (12)	C16—N3—C15—O5	168.82 (11)
C2—N1—C1—N2	−6.03 (19)	C16—N3—C15—N4	−13.53 (18)
C1—N1—C2—O2	−90.65 (15)	C15—N3—C16—O6	−83.84 (15)
C1—N1—C2—C3	150.12 (12)	C15—N3—C16—C17	156.78 (12)
C1—N1—C2—C4	30.96 (17)	C15—N3—C16—C18	37.46 (16)
O2—C2—C3—F2	174.10 (10)	O6—C16—C17—F5	57.31 (14)
N1—C2—C3—F2	−62.89 (14)	N3—C16—C17—F5	179.58 (10)
C4—C2—C3—F2	54.76 (14)	C18—C16—C17—F5	−62.96 (14)
O2—C2—C3—F3	−65.29 (13)	O6—C16—C17—F7	178.24 (10)
N1—C2—C3—F3	57.72 (14)	N3—C16—C17—F7	−59.49 (13)
C4—C2—C3—F3	175.37 (10)	C18—C16—C17—F7	57.97 (14)
O2—C2—C3—F1	54.03 (14)	O6—C16—C17—F6	−62.40 (13)
N1—C2—C3—F1	177.05 (11)	N3—C16—C17—F6	59.87 (13)
C4—C2—C3—F1	−65.30 (14)	C18—C16—C17—F6	177.33 (10)
O2—C2—C4—C5	−51.86 (14)	O6—C16—C18—C19	−54.09 (13)
N1—C2—C4—C5	−176.67 (10)	N3—C16—C18—C19	−178.11 (10)
C3—C2—C4—C5	67.14 (14)	C17—C16—C18—C19	66.20 (14)
O2—C2—C4—C8	70.19 (12)	O6—C16—C18—C22	66.94 (13)
N1—C2—C4—C8	−54.63 (14)	N3—C16—C18—C22	−57.08 (13)
C3—C2—C4—C8	−170.82 (11)	C17—C16—C18—C22	−172.77 (10)
C6—O4—C5—O3	−1.83 (18)	C20—O8—C19—O7	−2.87 (18)
C6—O4—C5—C4	−179.41 (10)	C20—O8—C19—C18	177.89 (10)
C2—C4—C5—O3	69.04 (17)	C22—C18—C19—O7	−49.13 (17)
C8—C4—C5—O3	−52.95 (17)	C16—C18—C19—O7	70.36 (16)
C2—C4—C5—O4	−113.42 (12)	C22—C18—C19—O8	130.10 (11)
C8—C4—C5—O4	124.58 (11)	C16—C18—C19—O8	−110.42 (12)
C5—O4—C6—C7	−84.19 (14)	C19—O8—C20—C21	−175.59 (11)
C1—N2—C8—C9	−155.05 (12)	C15—N4—C22—C23	−159.94 (12)
C1—N2—C8—C4	−32.65 (17)	C15—N4—C22—C18	−34.77 (16)
C5—C4—C8—N2	180.00 (10)	C19—C18—C22—N4	177.29 (10)
C2—C4—C8—N2	55.00 (13)	C16—C18—C22—N4	55.21 (13)
C5—C4—C8—C9	−59.74 (14)	C19—C18—C22—C23	−60.28 (14)
C2—C4—C8—C9	175.25 (11)	C16—C18—C22—C23	177.64 (10)
N2—C8—C9—C10	−116.25 (13)	N4—C22—C23—C24	−143.56 (12)
C4—C8—C9—C10	124.37 (12)	C18—C22—C23—C24	95.07 (14)
N2—C8—C9—C14	61.58 (15)	N4—C22—C23—C28	33.85 (16)
C4—C8—C9—C14	−57.80 (15)	C18—C22—C23—C28	−87.53 (15)
C14—C9—C10—C11	−0.64 (18)	C28—C23—C24—C25	0.19 (19)
C8—C9—C10—C11	177.25 (11)	C22—C23—C24—C25	177.67 (12)
C9—C10—C11—C12	1.40 (19)	C23—C24—C25—C26	−0.9 (2)
C10—C11—C12—F4	178.09 (11)	C24—C25—C26—F8	−178.72 (11)
C10—C11—C12—C13	−1.1 (2)	C24—C25—C26—C27	1.0 (2)
C11—C12—C13—C14	0.1 (2)	F8—C26—C27—C28	179.41 (11)
F4—C12—C13—C14	−179.12 (11)	C25—C26—C27—C28	−0.3 (2)
C12—C13—C14—C9	0.7 (2)	C26—C27—C28—C23	−0.5 (2)
C10—C9—C14—C13	−0.43 (19)	C24—C23—C28—C27	0.53 (19)
C8—C9—C14—C13	−178.30 (12)	C22—C23—C28—C27	−176.91 (12)

### Hydrogen-bond geometry (Å, °)

**Table:**

*D*—H···*A*	*D*—H	H···*A*	*D*···*A*	*D*—H···*A*
O2—H2···O9^i^	0.889 (15)	1.860 (16)	2.7482 (14)	176.2 (15)
O9—H9A···O3^ii^	0.91 (2)	1.90 (2)	2.8026 (15)	169.8 (18)
O6—H6···O10^iii^	0.893 (16)	1.788 (16)	2.6770 (14)	173.5 (15)
N3—H3A···O10^iii^	0.823 (14)	2.583 (15)	3.0737 (16)	119.6 (12)
N4—H4A···O5^iii^	0.904 (14)	1.900 (15)	2.8010 (14)	174.8 (13)
N1—H1A···O1^iv^	0.828 (14)	2.109 (14)	2.9235 (15)	167.9 (13)
N2—H2A···O5^v^	0.906 (14)	1.958 (15)	2.8395 (14)	163.8 (13)
O9—H9B···O1^vi^	0.80 (2)	2.00 (2)	2.7443 (15)	155 (2)
O10—H10A···O9^vii^	0.861 (17)	1.925 (17)	2.7833 (15)	175.4 (16)
O10—H10B···O7^viii^	0.82 (2)	2.07 (2)	2.8685 (14)	166 (2)

Symmetry codes: (i) *x*, *y*−1, *z*; (ii) −*x*, −*y*+1, −*z*+1; (iii) −*x*+1, −*y*+2, −*z*+2; (iv) −*x*+1, −*y*, −*z*+1; (v) −*x*+1, −*y*+1, −*z*+2; (vi) −*x*+1, −*y*+1, −*z*+1; (vii) −*x*+1, −*y*+2, −*z*+1; (viii) *x*+1, *y*, *z*.
